# Responses of soil bacterial communities and maize yields to sulfur application across four soil types

**DOI:** 10.3389/fmicb.2024.1329938

**Published:** 2024-03-13

**Authors:** Siqi Dong, Bing Zhang, Zhao Wang, Xue Zhou, Qiang Gao

**Affiliations:** Key Laboratory of Sustainable Utilization of Soil Resources in the Commodity Grain Bases of Jilin Province, College of Resource and Environmental Sciences, Jilin Agricultural University, Changchun, China

**Keywords:** soil type, sulfur rates, bacterial community structure, structural equation modeling, network

## Abstract

**Introduction:**

This study assessed the effects of S application on maize yields and soil bacterial communities across four sites with different soil types and three S application rates (0 kg ha^-1^, 30 kg ha^-1^, and 90 kg ha^-1^).

**Methods:**

Changes in soil properties, bacterial community diversity, structure, and their contributions to maize production were evaluated post-S application treatments.

**Results:**

(1) S application decreased soil pH, increased available sulfur (AS), and boosted maize yields in all soil types. (2) Reduced Chao1 and Shannon diversity indices were observed in black soil after S application. (3) Bacterial community structure was significantly affected by S application, except in sandy soil, impacting key stone taxa abundance. (4) Black soil showed higher sensitivity to S application due to less stable bacterial community structure. (5) Soil physicochemical indicators altered by S application, such as AS and pH, mediated bacterial diversity, influencing maize yield. Organic matter (OM) had the most significant direct positive effect on yield, followed by AS and bacterial community diversity.

**Discussion:**

This study emphasizes the impact of S application on soil properties and bacterial communities in diverse soil types. Understanding these mechanisms can guide precision S application practices for maize yield regulation.

## 1 Introduction

Sulfur (S) was another essential nutrient for plants, following nitrogen (N), phosphorus (P), and potassium (K). It serves as a crucial mineral nutrient, playing a vital role in regulating and controlling plant growth and development (Takahashi et al., 2011; Romero et al., 2014). S has multiple physiological and biochemical functions in plant growth. It is an integral element in amino acids and proteins, a foundational component of many cofactors and prosthetic groups, and an important structural substance of the chloroplast membrane and participates in redox reactions (Eriksen et al., 2002). S scarcity in soil has become increasingly pervasive all over China, as a result of the cautious use of S fertilizer on crops and increasing crop yields in recent years (Ramette, 2007; Wang et al., 2008). Excessive and imbalanced application of nitrogen, phosphorus, and potassium fertilizers, along with the utilization of pesticides containing S, have obscured deficiencies of S in the soil over an extended period. Decreasing the usage of fertilizers containing S, such as heavy calcium and superphosphate, also results in a decreasing amount of S nutrients available from the soil for crops, as a result of the maturity of the fertilizer manufacturing process and the ongoing optimization of the fertilizer structure (Li et al., 2017). As a result, more than 20% of the soil is in a potential sulfur-deficient state, which brings serious problems for agricultural production. When the supply of AS in the environment is insufficient to meet the needs of plant growth and development, can hinder the absorption of essential nutrients like as carbon and nitrogen, leading to disruption in protein biosynthesis, chlorophyll levels, and ultimately reducing crop yield (Capaldi et al., 2015). As far as Jilin Province, the soil AS was 27.2% below 12 mg kg^−1^, 20.7% was 12–16 mg kg^−1^, 12.2% was 16–20 mg kg^−1^, and more than 60% of the soil had a S deficiency (Liu, 2021). As an inevitable consequence of insufficient supply, crop yields were restricted. Therefore, more attention should paid to S, especially in the farmland ecosystem.

More than 95% of total sulfur (TS) in the soil environment forms combining with organic molecules, which include soil organic matter and microbial biomass. Inorganic S usually only accounts for a small proportion of the total amount. However, organic S must be mineralized to SO_4_^2−^ in order to be absorbed by the plant (Kertesz and Frossard, 2015; Anantharaman et al., 2018). S compounds in the soil undergo a series of transformations, including the conversion of sulfide into sulfate, immobilization of inorganic sulfate and organic S compounds into microbial biomass and OM, and the mineralization of soil organic S. These processes are predominantly mediated by microorganisms, especially bacteria (Ma et al., 2020). Besides the S cycle, soil microorganisms significantly influence the regulation of chemical cycles during the processes of biological succession in farmland ecosystems (Qi et al., 2021). Prior independent research has demonstrated that the application of sulfur resulted in substantial alterations to the soil bacterial community structure in both black and sandy soil (Samples collected in 2018; Dong et al., 2022). Overall, further field experiments are required to validate the correlation between the structure of microbial communities and the quantity of added sulfur.

To fully understand the effects of sulfur application on soil bacterial communities across various soil types and investigate the influence of soil physical and chemical properties, as well as microbial properties, on corn yield, we collected soil samples from four different soil types (black, sandy, saline, and dark brown soil). The aim was to comprehensively assess the impact of sulfur application on soil bacterial diversity and community structure in these diverse soil types. Additionally, we employed structural equation modeling (SEM) to explore the coupling effect of soil physicochemical properties on crop yield.

## 2 Materials and methods

### 2.1 Field sites and sample collection

In 2017, a field experiment was conducted at four sites: Sankeshu (43°20′N, 124°00′E), Fujia (43°21′N, 124°05′E), Helong (42°45′N, 129°21′E), and Tongyu (44°36′N, 123°04′E) in the Jilin Province of China. All sites had a temperate monsoon climate but a different soil type. Based on the soil classification system, the soil samples collected from Sankeshu, Helong, Fujia, and Tongyu were classified into black, dark brown, sandy, and saline soil, respectively. Annual precipitation at the four sites were780.3, 542.4, 732.5, and 350 mm, respectively. Full details and field information for each site are listed in Supplementary Table S1. Research plots were established to assess the S (ammonium sulfate) application rates on maize yield and soil bacterial community. Three S application rates were tested: 0 kg S ha^−1^(S0), 30 kg S ha^−1^ (S30), and 90 kg S ha^−1^ (S90). There were three replicates for each application rate, resulting in nine plots per site, structured in a random block design. The total number of plots for all sites was 36. The fertilizer application rates are summarized in Supplementary Table S2. Maize was sown in May with a planting density of 65,000 plants per hectare and harvested in October.

Soil samples were collected in September 2019. Five maize plants were chosen at random in each plot. All samples underwent a 2 mm sieving process to remove roots and other debris. Subsequently, five samples were pooled to generate a singular mixed rhizosphere soil sample for each plot (Azeem et al., 2020; Li et al., 2021; Yu et al., 2021). Subsamples designated for property analysis were stored at −20°C, while those allocated for DNA extraction were stored at −80°C.

### 2.2 Soil chemical properties and maize yield determination

The maize yields were measured at the harvesting stage of maize, and the number of plants, ears, and total fresh weight of maize ears within the yield area (18 m^2^) was recorded (Yu et al., 2019). The yields were calculated by 14% moisture content (Yu et al., 2019). Soil pH was determined using a pH meter at a soil: water ratio of 1:2.5. The soil organic matter (OM), available nitrogen (AN), available phosphorus (AP), and available potassium (AK) were analyzed by the potassium dichromate volumetric, diffusion method, the Olsen method, and the ammonium acetate extraction flame photometry method, respectively. The AS content was extracted using KH_2_PO_4_ and quantified using an Inductive Coupled Plasma Emission Spectrometer (ICP).

### 2.3 Illumina MiSeq high-throughput sequencing and data analysis

Soil DNA was extracted using the Fast DNA SPIN extraction kits (MP Biomedicals, Santa Ana, CA, United States), according to the manufacturer’s guidelines, with a negative control included.

The V3-V4 region of the 16S rRNA gene was amplified using the forward 338F (5′-ACTCCTACGGGAGGCAGCA-3′) and reverse 806R (5′-GGACTACHVGGGTWTCTAAT-3′) primers. Unique 7-bp barcodes, specific to each sample, were integrated into the primers for multiplex sequencing. PCR amplicons were purified utilizing Agencourt AMPure Beads from Beckman Coulter, located in Indianapolis, IN. Quantification was carried out employing the PicoGreen dsDNA Assay Kit provided by Invitrogen in Carlsbad, CA, United States. Following individual quantification, equal amounts of amplicons were mixed, and pair-end 2 × 300 bp sequencing was conducted using the Illlumina MiSeq platform and MiSeq Reagent Kit v3 at Shanghai Personal Biotechnology Co., Ltd. in Shanghai, China.

The sequence data were processed using the Quantitative Insights into Microbial Ecology (QIIME2 2019.4), following a previously established protocol (Bolyen et al., 2019). After removing barcode and primer sequences, a quality filtering step was executed to eliminate ambiguous sequences that did not meet the quality criteria. UCHIIME algorithms were utilized to remove chimeric sequences. The resulting valid sequences were subsequently assigned for taxonomic classification and a representative sequence at the ASV level was chosen with default parameters, specifically targeting a 98% identity level. The analysis of alpha indexes, including Chao1, Shannon, Observed species, and Goods-coverage indices, was conducted using the QIIME2 software.

### 2.4 Statistical analysis

Statistical analyses were performed using SPSS Statistics for Windows v 25.0 (IBM, Armonk, NY, United States). Differences in soil properties, maize yield, alpha diversity, and modularity index were determined by one-way ANOVA followed by Duncan’s multiple range test (*p* < 0.05). Two-way ANOVA was used to evaluate the effects of interactions between S fertilization rates and the different soil types on maize yield and soil properties (Chen et al., 2021).

The visualization of differences in microbial community composition across all soil samples (four soil types) was carried out using NMDS analysis with Bray–Curtis distance matrices, utilizing the “vegan” package for R (version 4.1.2). The NMDS analysis was considered reliable when the Stress value was less than 0.2. Furthermore, to assess the differences in soil microbial community composition between different S application treatments within each soil type, Analysis of Similarities (ANOSIM) was performed.

Network analysis was employed to investigate the co-occurrence patterns of rhizosphere bacterial communities across four distinct soil type. We calculated the Spearman correlations between ASVs with all the samples (four soil types) and identified strongly associated ecological clusters (modules). To construct the network, only robust (Spearman’s *r* > 0.6 or *r* < −0.6) and statistically significant (*p* < 0.05) correlations were taken into consideration, utilizing the “psych” package for R (version 4.1.2) (Jiao et al., 2019). This approach enabled us to concentrate specifically on the ASVs exhibiting robust co-occurred, increasing the likelihood of interactions among them, and promoting consistent module patterns across each soil type. The main modules in the network of each soil type were visualized using the interactive platform Gephi 0.9.2 (Zhang Y. et al., 2021). Seven network level topological characteristics were calculated.

Computed the total abundance of all genera in the primary network *n* modules and utilized *Z*-scores to standardize the cumulative abundance of each module, thus establishing it as the module’s relative abundance. The differences of genera present within the module were determined by one-way ANOVA followed by Duncan’s multiple range test (*p* < 0.05).

To investigate the relationship between soil physicochemical properties, bacterial communities, and maize yield, we utilized structural equation modeling (SEM), implemented through the R package lavaan. This approach enabled us to evaluate both direct and indirect effects of soil properties and bacterial communities on crop productivity. The PC1 axis from Principal Component Analysis (PCA) were selected as the parameter for bacterial community structure, as per the ASV table. Typically, the approximate root mean square error and chi-square value (χ^2^). We employed the Akaike Information Criterion (AIC) as a comprehensive measure to assess the model’s goodness of fit. The best-fitting SEM was determined based on the chi-square test (with a significance level of *p* > 0.05) and the root mean square approximation error (RMSEA<0.2). All analyses were conducted using R (version 4.1.2).

The predicted the functional attributes of bacterial community, we utilized the Kyoto Encyclopedia of Genes and Genomes (KEGG) database, accessible through the following link: http://www.genome.jp/kegg/. The prediction was executed using the Phylogenetic Investigation of Communities by Reconstruction of Unobserved States (PICRUSt2) tool (Version 2.2.0), available at: https://github.com/picrust/picrust2. PICRUSt2 estimates the functional composition of the bacterial community based on the proportional representation of marker gene sequences within the samples. The present study examines the abundance of Pathway level 3 through a sample heatmap analysis conducted using the R programming language (Douglas et al., 2020).

## 3 Results

### 3.1 Effect of S fertilization on maize yield and soil physicochemical properties

**Figure 1:**
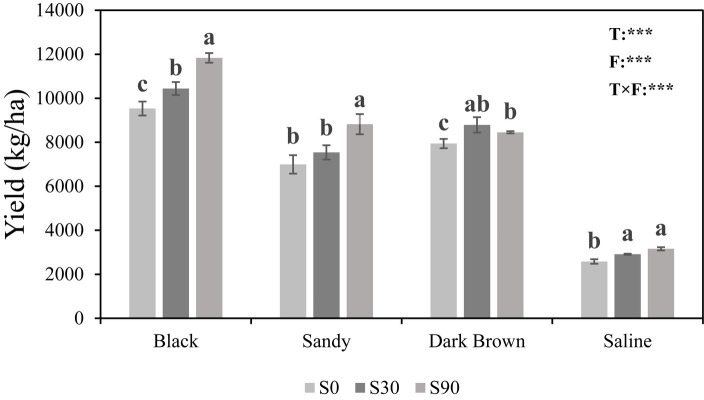
Histogram showing maize yields in four soils in response to Sulfur (S) application at three rates (0, 30, and 90 kg S ha^−1^). Different letters indicate significant differences between treatment groups in each soil type (*p* < 0.05). For the ANOVA results: ^*^, ^**^, and ^***^ indicate that there are significant differences for each factor at the levels of *p* = 0.05, *p* = 0.01, and *p* = 0.001. T, Soil types; F, Fertilizer; and T × F, Soil types × fertilizer.

Regardless of soil type, the application of S led to higher maize yields compared to the S0 treatment, where no S was added. The highest maize yield was found in black soil and the lowest in saline soil under the same S application rate. Two-way ANOVA showed both soil types (*F* = 960.42; *p* < 0.001) and fertilization (*F* = 54.81; *p* < 0.001) have a very significant impact on maize yield, and the interaction between these two also has a significant impact (*F* = 8.36; *p* < 0.001) (Figure 1).

pH decreased significantly with the increase of S application, except for sandy soil (Table 1), and the changes in pH were consistent among other three soil types. AS gradually increased with S application rates and reached its peak in S90 treatment. There was no significant variations observed in other soil physicochemical parameters, including AN, AP, AK, and OM. Based on the findings from the Two-way ANOVA analysis, the rate of S application demonstrated a significant impact on the pH and AS, while the type of soil exhibited a significant relationship with all of the chemical properties of the soil.

**Table 1:** Soil characteristics under application of S application.

Soil types	Sample	pH	AP (mg/kg)	AK (mg/kg)	AN (mg/kg)	OM (g/kg)	AS (mg/kg)
Black	S0	5.78 ± 0.07^a^	51.3 ± 4.82^a^	185.1 ± 7.93^a^	128.7 ± 3.25^a^	21.71 ± 0.79^a^	13.3 ± 1.31^b^
	S30	5.70 ± 0.05^ab^	50.0 ± 1.68^ab^	187.4 ± 3.83^a^	128.3 ± 1.72^ab^	21.70 ± 2.28^a^	18.3 ± 1.03^a^
	S90	5.62 ± 0.02^b^	48.4 ± 6.60^b^	182.2 ± 8.45^a^	126.5 ± 2.18^ab^	21.80 ± 2.98^a^	21.3 ± 2.87^a^
Sandy	S0	5.57 ± 0.03^a^	33.0 ± 2.46^ab^	157.5 ± 1.30^a^	66.9 ± 2.73^a^	15.67 ± 0.02^a^	11.8 ± 1.03^b^
	S30	5.53 ± 0.11^a^	33.9 ± 1.38^a^	161.8 ± 2.87^a^	66.0 ± 3.34^ab^	16.05 ± 0.10^a^	15.7 ± 0.24^a^
	S90	5.50 ± 0.08^a^	34.2 ± 0.76^a^	162.3 ± 4.17^a^	65.5 ± 4.49^b^	15.77 ± 0.31^a^	17.5 ± 1.08^a^
Dark brown	S0	5.58 ± 0.03^a^	27.1 ± 2.97^a^	167.9 ± 1.08^a^	90.9 ± 1.15^a^	12.44 ± 1.52^a^	12.7 ± 1.31^b^
	S30	5.48 ± 0.03^b^	25.9 ± 2.38^a^	169.7 ± 4.17^a^	90.7 ± 2.76^a^	12.49 ± 0.87^a^	18.0 ± 1.08^a^
	S90	5.36 ± 0.052^c^	25.8 ± 1.70^a^	168.4 ± 0.36^a^	90.4 ± 5.57^a^	12.62 ± 0.50^a^	20.5 ± 3.08^a^
Saline	S0	8.64 ± 0.07^a^	35.9 ± 0.61^a^	203.2 ± 5.92^a^	40.7 ± 4.08^a^	10.29 ± 1.19^a^	11.0 ± 0.82^c^
	S30	8.33 ± 0.02^b^	36.0 ± 0.28^a^	202.2 ± 5.56^a^	38.0 ± 1.75^ab^	10.39 ± 1.95^a^	14.3 ± 0.62^b^
	S90	8.03 ± 0.04^c^	36.8 ± 0.73^a^	198.3 ± 7.99^a^	37.3 ± 1.86^ab^	10.43 ± 1.16^a^	17.2 ± 1.31^a^
ANOVA							
T		^***^	^***^	^***^	^***^	^***^	^**^
F		^***^	ns	ns	ns	ns	^***^
T × F		ns	ns	ns	ns	ns	ns

In the results of the ANOVA, different letters indicate significant differences treatment in each soil types (*p* < 0.05). ^*^, ^**^, and ^***^ indicate that there are significant differences between the treatments at the levels of *p* = 0.05, *p* = 0.01, and *p* = 0.001, and ns indicates that the difference is not significant (*p* > 0.05). T, Soil types; F, Fertilizer; T × F, Soil types × fertilizer.

### 3.2 Effects of S application on bacterial alpha diversity

**Figure 2:**
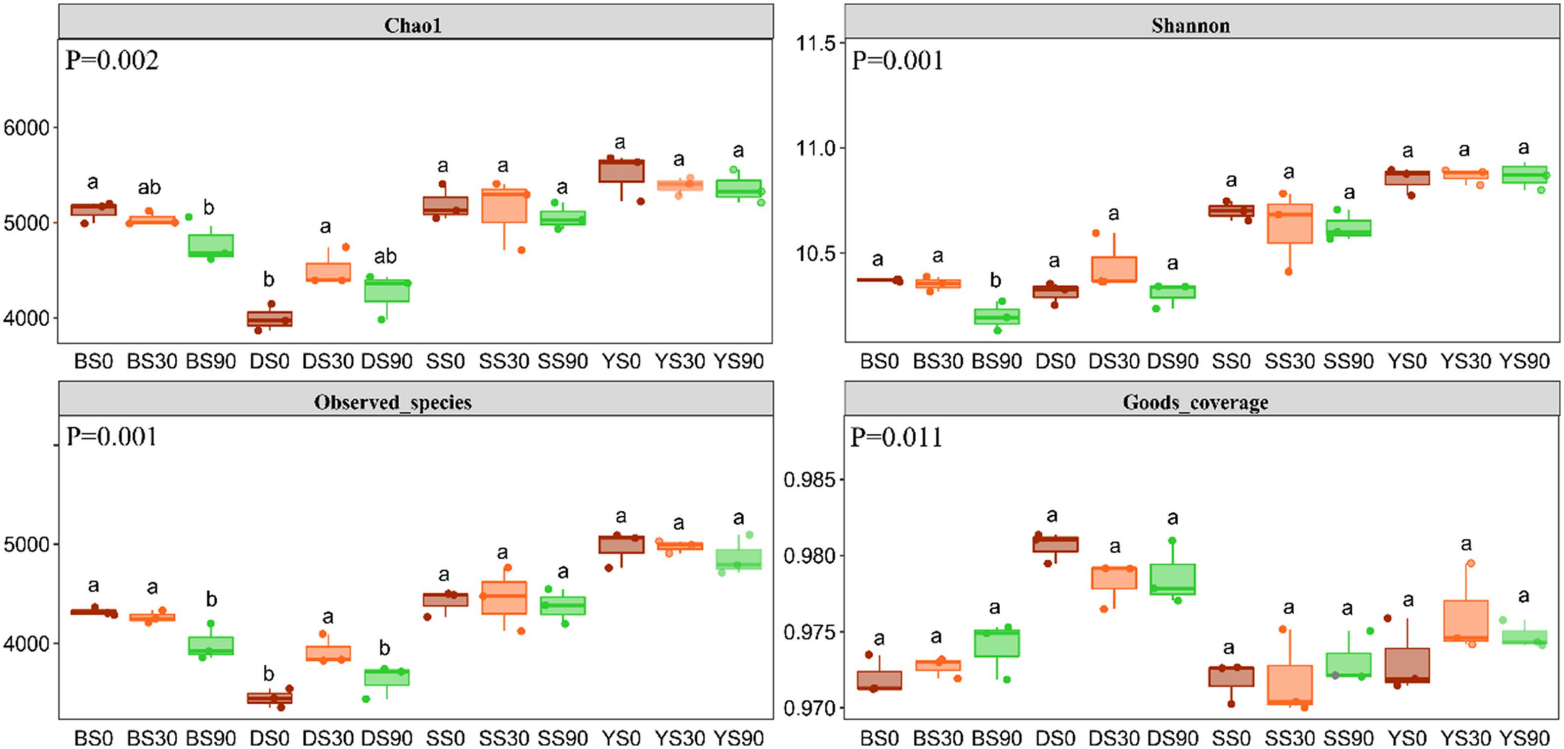
Alpha diversity indices of soil microbial community under three rates S application (0, 30, and 90 kg S ha^−1^). Different letters indicate significant differences for each factor (*p* < 0.05). *p* value of inter group population differences obtained from Kruskal Wallis non parametric test. B, Black soil; D, Dark brown soil; S, Sandy soil; and Y, Saline soil.

The Good’s coverage for the observed bacterial ASVs was 97.47 ± 0.29% (mean ± s.e), indicating a high probability of sequence detection in the samples. The Chao1 and Shannon indexes were used to evaluate the total number of ASVs contained in the samples and estimate the diversity of microbial communities. The bacterial Chao1 diversity index was highest in the S0 treatment except for dark brown soil, and higher Chao 1 index was observed in all treatments of black soil. Bacterial alpha diversity decreased with S application in black soils, while it increased in S30 treatments in dark brown soils. However, alpha diversity in saline and sandy soils was not impacted by S application rates. Overall, the alpha diversity appeared to be highest in saline soil, despite this soil yielding the lowest maize biomass (Figure 2).

### 3.3 Effects of S application on bacterial community stability in maize soils

**Figure 3:**
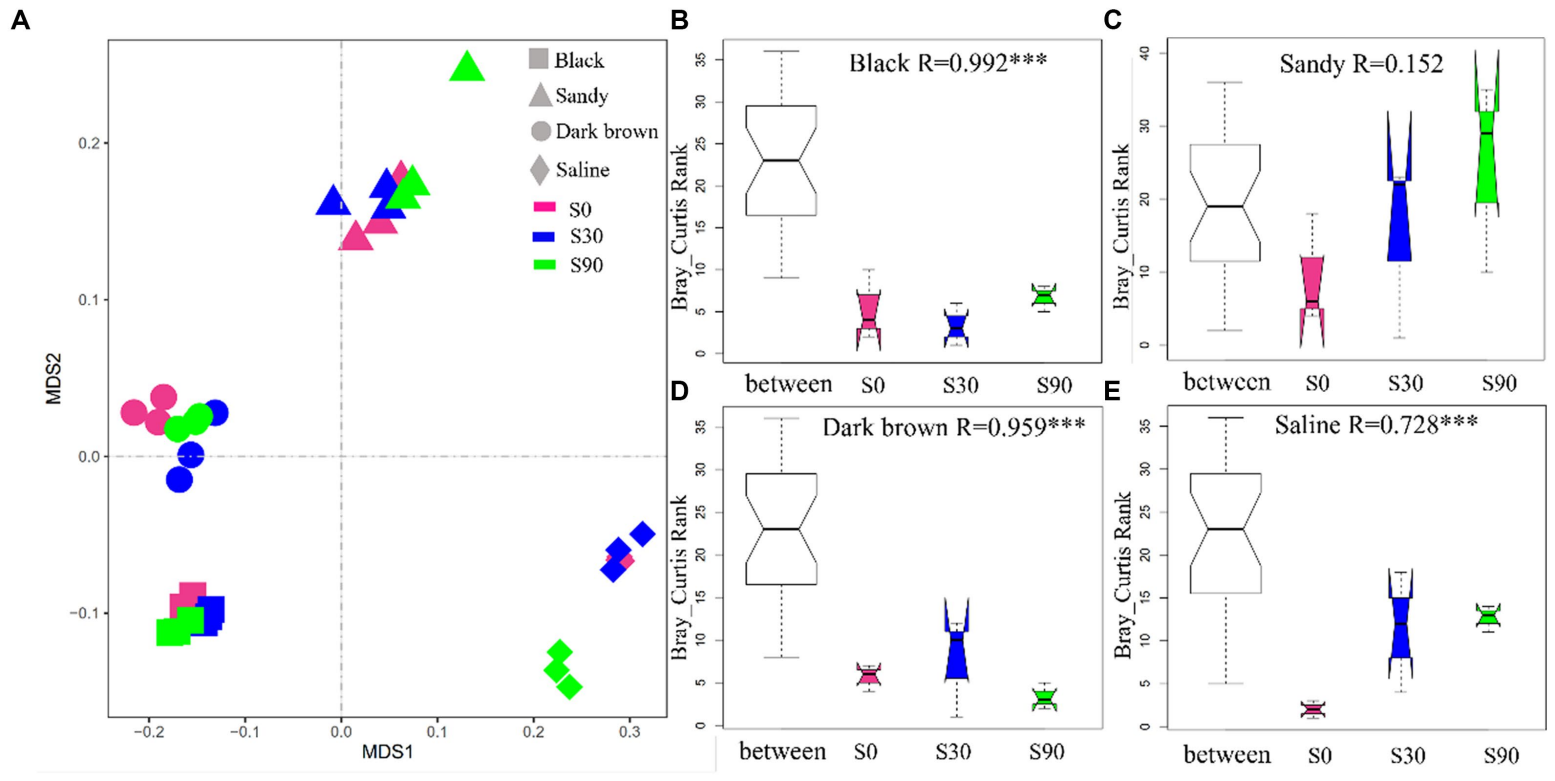
Non-metric multidimensional scaling ordination based on Bray-Curtis distances comparing the composition of soil bacterial communities **(A)**. ANOSIM was used to test the difference of soil bacterial between different S application treatments within the same soil type **(B–E)**.

The NMDS analysis of all the soil samples (four soil types) showed that soil type formed distinct clusters in the coordinate (Figure 3A), and the difference of soil bacterial community between S application treatments within the same soil type was further revealed by ANOSIM, which considers both within and between treatment variance. The ANOSIM results indicated significant changes (*p* < 0.05; Figure 3B) between different S application treatments excluding sandy soil, which indicates that S application significantly affected the community structure of soil bacteria. In addition, according to the observed *R* value of ANOSIM analysis, among the four soils, S application had a greater impact on the bacterial community of back soil. This is partly attributed to the relatively lower variability between samples of the same treatment within black soils.

To assess the influence of S application on the soil bacterial community, a correlation network was constructed, incorporating the identified soil bacterial. We further defined nodes with more than 10 edges as network active centers, and three ecological clusters (modules) that strongly co-occurring was revealed, which play an active role in regulating the interactions between communities.

**Figure 4:**
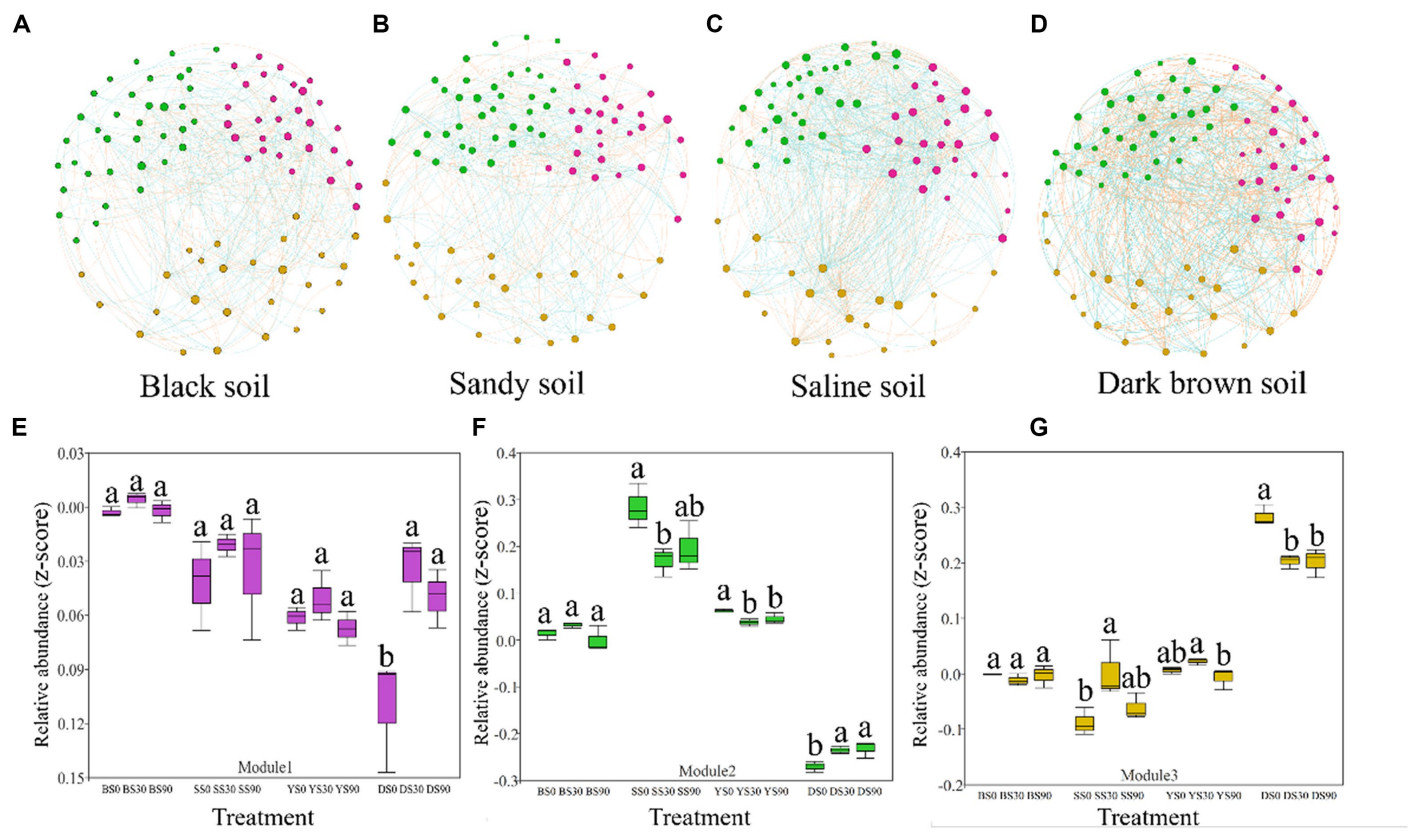
Co-occurrence networks of the bacterial communities in the maize rhizosphere were constructed across four soil types based on correlation analysis. A connection in the network represents a robust (Spearman’s *r* > 0.6 or *r* < −0.6) and statistically significant (*p* < 0.05) correlation. Each node in the graphical representation corresponds to a specific genus, with node size indicating the number of connections (degree). The node colors correspond to different categories: pink nodes represent module 1, green nodes represent module 2, and yellow nodes represent module 3 **(A–D)**. The red and blue colors of each connection between two nodes represent positive and negative relationships, respectively, based on Spearman’s correlation coefficients. Each edge in the network indicates a strong and statistically significant correlation between two nodes, with the thickness of the edge proportional to the value of the Spearman’s correlation coefficient. The relative abundance (*z*-score of accumulated abundance) of microbial clusters across treatments in the four types of soil is shown in **(E–G)**. Different letters indicate significant differences between sulfur application rates (0, 30, and 90 kg S ha^–1^) for each soil type [Black (B), Sandy (S), Saline (Y), and Dark Brown (D)] as determined by ANOVA (*p* < 0.05).

The network analysis results revealed that bacterial network density was highest in saline soil, followed by dark brown and sandy soil, and lowest in black soil (fewer nodes result in more edges). However, upon compared network-level topological characteristics, it was observed that the dark brown and saline soil networks exhibited significantly higher average degree and clustering coefficient values compared to the black and sandy soil networks. These findings suggest that the networks of dark brown and saline soil were more interconnected and displayed stronger relationships among their components, and the bacterial community structure of black soil was less stable. This information is visually depicted in Figures 4A–D and is also available in Supplementary Table S3.

S application regulates the co-occurrence of bacterial networks by changing the abundance of keystones in the module. The S application in dark brown soil significantly increased the abundance of module 1. The S application in sandy and saline soil reduced the abundance of module 2, but the results were opposite in dark brown soil. The results in module 3 were consistent with the changes in abundance in module 2, except that the abundance of S90 treatment in saline soil was lower than that of S0 and S30 treatment (Figures 4E–G). We found that in the modules 1, 2, and 3, the more abundant genera were all from *Patescibacteria*, *Actinobacteria*, and *Proteobacteria* (co-trophic groups) (Supplementary Table S6).

### 3.4 Effect of S fertilization-driven soil properties on maize yields

**Figure 5:**
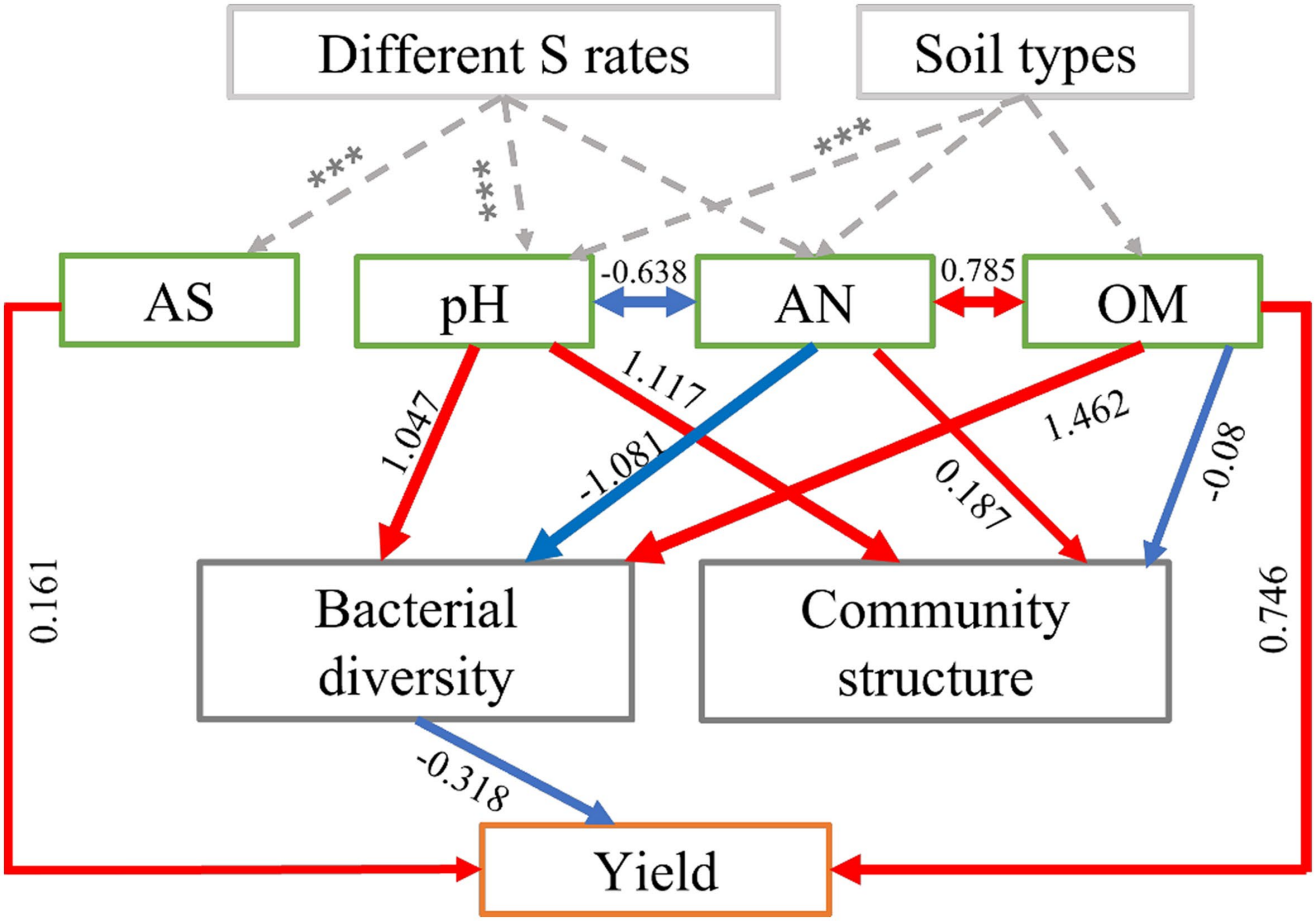
Structural equation model (SEM) showing the direct and indirect effects of soil physicochemical properties (pH, OM, AN, and AS) and bacterial community diversity and structure on maize yield in S-amended treatments and four soil types. In the diagram, red arrows represent positive relationships (*p* < 0.05), while blue arrows represent negative relationships (*p* < 0.05). The numbers displayed on the arrows, which serve as standardized path coefficients, indicate the percentage of variance explained by the predictor variables. The width of each arrow reflects the strength of the standardized path coefficient. Soil organic matter content (OM), available nitrogen (AN), and available sulfur (AS).

We employed structural equation modeling (SEM) to assess both the direct and indirect influenced of soil physicochemical properties and bacterial communities on the yields of maize. The model fit the data well (χ^2^ = 11.502, *p* = 0.243, AIC = 79.792, SMSEA = 0.152; Supplementary Table S4). The results showed that S application and soil types had a significantly impact on the physicochemical properties of the soil (such as pH and AS), these factors exerted control over the maize yield either independently or in combination, while also influencing the soil bacterial communities, including their structure and diversity, which in turn mediated the impact on maize yield (Figure 5). Overall, the OM (path coefficient = 0.746) had the most significant positive and direct impact on maize yields, followed by soil AS (0.161) (Figure 5). Bacterial diversity (−0.318) had direct and negative effects on crop yields (Figure 5). In addition, soil pH and AN had significantly higher indirect effects than other driving factors. S application directly affects the changes in pH, which likely mediates the bacterial diversity and leads to the final regulation of maize yield.

### 3.5 Effect of S application on potential functions of the sulfate reduction

**Figure 6:**
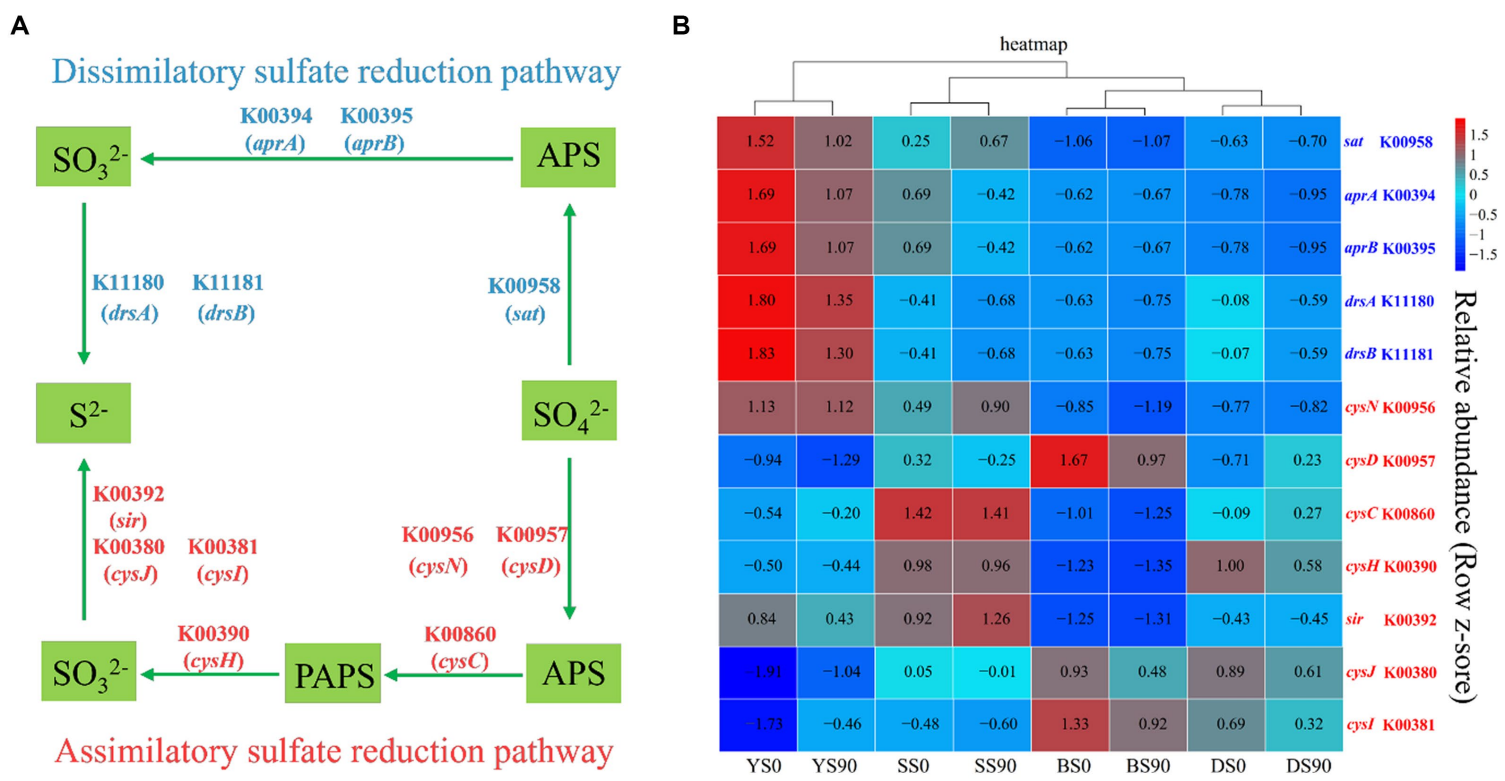
The sulfate reduction pathways and associated gene abundances. **(A)** The dissimilatory and assimilatory sulfate reduction pathways. **(B)** Relative abundances of PICRUSt predicted genes relevant with S metabolism across the S application (0, 90 kg S ha−^1^) in four soils [Black (B), Dark Brown (D), Sandy (S), and Saline (Y)]. The red and blue fonts indicate genes relevant with assimilatory and dissimilatory sulfate reduction pathways, respectively.

The sulfate reduction pathway encompasses both assimilatory and dissimilatory processes, which involve the reduction of sulfate to sulfide. Most prokaryotes with sulfate reducing ability are bacteria. Five genes control the three sequential enzymatic steps in the dissimilatory sulfate reduction pathway (Figure 6A). Conversely, the assimilatory sulfate reduction pathway comprises four steps controlled by five enzymes, encompassing a total of seven (Figure 6A). In this study, PICRUSt was utilized to predict the relative gene abundances related to dissimilatory sulfate reduction. In all four types of soil, S application reduced the abundance of five dissimilatory sulfate reduction genes, including sulfate adenylyltransferase (*sat*), adenylylsulfate reductase subunit A (*aprA*), adenylylsulfate reductase subunit B (*aprB*), dissimilatory sulfite reductase subunit alpha (*drsA*), and dissimilatory sulfite reductase subunit beta (*drsB*). The difference lies in the higher abundance of sat in sandy soil under the S90 treatment. The abundance of most assimilatory sulfate reduction genes decreased with S application (except for sulfate adenyltransferase (*cysN*) and sulfite reductase (*sir*) in sandy soil, adenylylsulfate kinase (*cysC*) and sulfate adenylyltransferase subunit (*cysD*) in dark brown soil, phosphoadenosine phosphosulfate reductase (*cysH*), sulfite reductase (NADPH) flavoprotein alpha-component (*cysJ*), and sulfite reductase (NADPH) hemoprotein beta component (*cysI*) in saline soil). Particularly in black soil, S application inhibited the abundance of all assimilatory sulfate reduction genes (Figure 6B; Supplementary Table S5).

## 4 Discussion

### 4.1 The responses of soil physicochemical properties and maize yield to S application

Our findings indicated that the application of S resulted in a significantly increase in maize yield (Figure 1). The highest yield was observed with the S90 treatment across various soil types, with the exception of dark brown soil, which the differences between the S30 and S90 treatments were not statistically significant. Our findings align with the results obtained reported by Cao et al. (2017), demonstrating that S application effectively enhanced maize yield. Specifically, Cao et al. (2017) reported that in dark brown soil, the optimal application rate for S was 80 kg ha^−1^, resulting in a significant increase in maize yield. Under uniform S application rates, maize yields exhibited variability, with the highest yield observed in black soil and the lowest in saline soil. The OM emerged as a significant underlying determinant, exerting an indirect influence on crop yields by fostering microbial biomass and enhancing soil enzyme activity (Luo et al., 2018). The deleterious effects of saline soil on plant physiology manifest through multiple mechanisms such as: osmotic stress, ion imbalances, perturbations in nutrient equilibria, metabolic dysregulation, and diminished cellular division rates, which collectively impinging upon overall crop productivity (Gharib et al., 2016; Hafez et al., 2020).

Soil physicochemical properties are important indexes to evaluate soil fertility and are affected by various factors. Previous research has confirmed that when S enters the soil, it undergoes oxidation by soil bacteria, such as *Thiobacillus*, converting it to sulfuric acid. This process leads to a reduction in soil pH, and increased S application exacerbates the decline in soil pH (Germida and Janzen, 1993; Du et al., 2020). In our investigation, while maintaining a consistent input of NPK fertilizers, we observed a gradual decreased in soil pH corresponding to an increase in S application (Table 1), aligning with the findings mentioned earlier. Furthermore, our findings revealed a positive correlation between S application and the AS, aligning with the findings of Sachiko (Masuda et al., 2016). The probable explanation for this phenomenon is that oil microorganisms primarily participate in the oxidation process of S application mainly affects the relative abundance of sulfate-reducing bacteria and the amount of AS (Masuda et al., 2016).

The present study builds upon the foundation established by our previous work (Dong et al., 2022), which examined similar research questions with a focus on two specific soil types. In the current study, we extend this investigation to encompass a broader scope, involving four distinct soil types and a longer experimental timeframe of 3 years. In our previous studies (Dong et al., 2022), we did observe a decrease in OM in black soil with S application. However, in this study, we did not observe any significant variations in OM due to S application (Table 1). This discrepancy could be attributed to the fact that this study was conducted over a longer timeframe. Although S application has been widely studied in relation to soil pH, AS, and its interaction with heavy metals such as cadmium. These observations have been reported in a range of studies (Skwierawska et al., 2008; Masuda et al., 2016; Li et al., 2019; Wu et al., 2019; Du et al., 2020). However, we acknowledge that the potential impact of S application on the content of OM has not been extensively documented in the literature. To address this, we are committed to monitoring and documenting the long-term changes in organic material content at our experimental site.

### 4.2 The response of soil bacterial diversity and community structure to S application

Soil microbes can facilitate a series of processes, including sulfur oxidation and reduction, in the soil to promote the conversion of S within the soil. The fertilizer application was likely to cause changes in the structure of soil bacterial communities, based on previous research (Li et al., 2017). According to Dong’s report, the Chao 1 index shown no significant difference among the S0, S30, and S90 treatments after 2 years of S application (Dong et al., 2022). However, the Shannon index of S0 treatment was significantly higher than that of S30 treatment in black soil. In congruence with this, in this study, we found that after 3 years of S application, the Chao1 and Shannon indexes of S0 treatment were significantly higher than S90 treatment in black soil (Figure 2). In sandy soil, Dong et al. (2022) noted a higher Chao1 index in S0 treatment compared to S90 treatment, while our current study revealed no significant differences in the Chao1 and Shannon indexes among the three treatments in sandy soil. We recognize that such variations might be influenced by factors that require longer-term continuous observation to establish consistent patterns. The context of soil dynamics, microbial interactions, and environmental conditions could contribute to the observed variability in results over time. Consequently, we plan to extend our observations beyond the current timeframe to gain a more robust understanding of these patterns. In this study, the highest diversity and abundance of bacteria were observed in saline soil, possibly attributed to prolonged salinization and S application reducing the soil pH. The soil pH reduction created a conducive environment for maize growth and offered increased nutrients for microorganisms. Therefore, the alpha diversity index of bacteria was higher. The application of S altered the soil’s chemical properties, consequently impacting the functions of soil microorganisms. The health and productivity of an ecosystem depend on a range of factors, including the number and types of bacteria and other microorganisms.

According to the NMDS analysis, it was concluded that the effect of different S application rates on bacterial community structure was significant in all soil types apart from sandy soil (Figures 3B–E). Vincent findings indicated that both soil and crop types significantly influence the structures of soil bacterial communities (Tardy et al., 2015), which was also confirmed by Santosh ANOSIM analysis showed that the difference caused by soil types was much greater than the amount and type of S application (Mohanty et al., 2013). Earlier studies had indicated that the type of soil plays a predominant role in shaping the structure of rhizosphere bacterial communities. Additionally, it has been observed that a singular application of chemical fertilizers has a limited impact on the overall composition of microbial communities, although it may influence the abundance of specific groups (De Brito Ferreira et al., 2017). The previous research also observed that the S application had the greatest effect on bacterial community structure in black and dark brown soils (Figures 3B,D). Co-occurrence network analysis has been used to explore the underlying mechanisms of bacterial communities in various habitats and to identify the key groups that have the greatest impact on microbial communities (Faust and Raes, 2012; Liu et al., 2020; Ji et al., 2021). Network stability is closely related to network complexity. As a result, the closely related microorganisms in this study are artificially segmented into a module, where species with high abundance are designated as keystones, which are thought to collectively carry out the majority of functions. S applications changed the abundance of the keystone. Keystone species are represented was shown in the Supplementary Table S6. In order to further confirm the reaction of microorganisms in different soils and verify ANOSIM results, bacterial network diagrams were analyzed. S application led to changes in the relative abundance of bacteria; there was no significant change in the relative abundance of module 1, and a relative increase in the abundance of module 2, especially for S30 in the dark brown soil and S90 in saline soil treatments. The relative abundance of module 3 decreased significantly in sandy and dark brown soil, but increased significantly in saline soil (Figure 4G). We also found that in the modules 1, 2, and 3, the more abundant genera were all from *Patescibacteria*, *Actinobacteria*, and *Proteobacteria* (co-trophic groups) (Supplementary Table S6), which have the functions of decomposing nutrients, promoting the decomposition of animal and plant residues and promoting the absorption of nutrients by the root system (Wang M. et al., 2021). Compared the network properties of four different types of soil, the number of nodes and connections in dark brown and saline soil was greater, the connections between nodes are more complex, and the molecular ecological network becomes more robust. The possible reason is that the content of organic matter in the soil is low and the rhizosphere effect of syntrophic bacteria is abundant, which strengthens the connection between bacterial communities. Conversely, the average degree of the black and sandy soil networks was smaller, indicating that the material information transfer efficiency between species is higher, but fewer connections and relationships. Therefore, they have a sensitive response to the outside world (Yu et al., 2021; Zhang H. et al., 2021). Because higher organic matter results in a weakened rhizosphere effect, thereby avoiding competition (Gustavo, 2022).

### 4.3 Investigating the direct and indirect effects of soil properties influenced by S fertilization on maize yields

Structural equation modeling analysis revealed that S application influenced soil physicochemical property, which in turn had direct or indirect effects on maize yields by impacting soil bacterial communities (Yu et al., 2019). The soil pH contents were the most important drivers controlling maize yields indirectly, through mediation of soil bacterial communities. In addition, AS and OM content directly affected maize yield (Figure 5). Microbial activities facilitate S oxidation and reduction reactions, which are pivotal in driving the soil sulfur cycle (Su et al., 2017; Wang Q. et al., 2021). Several investigation shaved demonstrated that fertilizers indirectly alter the soil bacterial community structures and other characteristics or directly affect crop production by affecting soil physicochemical and microbial properties. Therefore, reasonable fertilization has great significance to improve crop yields and maintain soil properties (Yu et al., 2016; Hou et al., 2022).

### 4.4 Predicted pathways of bacterial communities in maize rhizosphere soil

At present, there is limited research on the prediction of functional characteristics of bacterial community structure in maize rhizosphere soil in response to sulfur fertilizer application. In this article, the use of PICRUSt2 expands upon the original PICRUSt1 approach for predicting community functional potential based on marker gene sequencing date. It is utilized to investigate the influence of S application on genes associated with sulfate reduction functional (Wang et al., 2022).

Sulfate was turned into adenosine phosphosulfate (APS) to 3′-phosphoadenylyl sulfate (PAPS) to sulfite to sulfide (Zhang et al., 2019). A total of five genes were detected to participate in the four-step reaction process of the dissimilatory sulfate reduction pathway. Firstly, sulfate was converted into ammonium persulfate (APS), and then APS was converted into sulfite. Finally, sulfite was transformed into sulfide (Zhang et al., 2019). A total of seven genes were detected to participate in the four-step reaction process of the assimilatory sulfate reduction. The genes K00394 (*aprA*), K00395 (*aprB*), K11180 (*drsA*), and K11181 (*drsB*) exhibit a negative trend with S application. This validates that the high application of S inhibited the dissimilatory sulfate reduction process (Li et al., 2022). While reports on the assimilatory sulfate reduction process are limited, it has been observed that assimilatory sulfate reduction occurs more frequently compared to dissimilatory sulfate reduction (Linz et al., 2018). The results of this study showed that more sulfate reducing genes were enriched in S0 treatment (*sat*, *aprA*, *aprB*, *drsA*, and *drsB*), which is consistent with the research results of Zhang H. et al. (2021).

## 5 Conclusion

In summary, according to the results of the present study, S application can notably alter soil physiochemical properties. With increased S application, soil pH gradually decreased and AS steadily increased. Compared to the other three soil types, black soil was more sensitive to the S application. In addition, network analysis showed that S application alters the abundance of keystone taxa in all four types of soil. The application of S reduced of most dissimilatory and assimilated sulfate reduction genes. S application and the type of soil, either together or independently, have an effect on both the properties of the soil and the structure of the bacterial community. The properties of the soil, along with the bacterial communities, directly and indirectly impacts on maize yields with S application.

## Data availability statement

The datasets presented in this study can be found in online repositories. The names of the repository/repositories and accession number(s) can be found here: BioProject, PRJNA893081.

## Author contributions

SD: Conceptualization, Data curation, Formal Analysis, Investigation, Methodology, Writing – original draft. BZ: Software, Writing – original draft. ZW: Software, Writing – original draft. XZ: Supervision, Validation, Writing – review & editing. QG: Supervision, Validation, Writing – review & editing.
