# Supplementary material for: Responses of soil bacterial communities and maize yields to sulfur application across four soil types
Source: Front Microbiol. 2024 Mar 13;15:1329938. doi: 10.3389/fmicb.2024.1329938 (PMC10965609; doi:10.3389/fmicb.2024.1329938)
Supplement: Supplementary file 1 [file Table_1.DOCX]

**Table S1.** Basic soil physical and chemical properties.

| Location | Soil Types | Abbreviation | pH | OM (g/kg) | AN  (mg/kg) | AP  (mg/kg) | AK  (mg/kg) | AS  (mg/kg) |
| --- | --- | --- | --- | --- | --- | --- | --- | --- |
| Sankeshu | Black | B | 6.63 | 23.2 | 106.6 | 38.4 | 191.3 | 13.2 |
| Fujia | Sandy | S | 5.77 | 16.2 | 61.7 | 25.0 | 160.9 | 11.3 |
| Helong | Dark brown | D | 5.93 | 13.1 | 76.9 | 18.6 | 170.6 | 12.6 |
| Tongyu | Saline | Y | 8.04 | 10.2 | 33.8 | 35.6 | 200.1 | 10.7 |

**Table S2.** Detailed fertilization processing.

|  |  | Pure nutrient input (kg/ha) | | |  |  |
| --- | --- | --- | --- | --- | --- | --- |
| Sample | S | | N | P | | K |
| S0 | 0 | | 210 | 90 | | 90 |
| S30 | 30 | | 210 | 90 | | 90 |
| S90 | 90 | | 210 | 90 | | 90 |

**Table S3.** Network-level topological features of the bacterial networks in the rhizosphere of maize across four soil types.

| Soil types | Node number | Edge  number | Average  degree | Network  diameter | Modularity | Clustering  coefficient | Average  path length |
| --- | --- | --- | --- | --- | --- | --- | --- |
| Black | 92 | 356 | 7.739 | 8 | 0.461 | 0.374 | 3.029 |
| Sandy | 91 | 354 | 7.780 | 7 | 0.517 | 0.451 | 2.935 |
| Saline | 79 | 618 | 15.646 | 8 | 0.289 | 0.540 | 2.645 |
| Dark Brown | 89 | 592 | 26.607 | 6 | 0.472 | 0.513 | 2.447 |

**Table S4.** DF=degrees of freedom, c^2^=chi-square (minimum function test statistic), RMSEA=root mean square error of approximation.

| Fit index | c^2^ | P | DF | RFI | NFI | IFI | RMSEA | AIC | BIC | ECVI |
| --- | --- | --- | --- | --- | --- | --- | --- | --- | --- | --- |
| Result | 11.502 | 0.243 | 9 | 0.838 | 0.931 | 0.984 | 0.152 | 79.792 | 88.520 | 3.959 |

**Table S5.** S cycle functional gene corresponds to KO.

| KO | Genes relevant with S | KO | Genes relevant with S |
| --- | --- | --- | --- |
| Dissimilated sulfate reducing gene | | Assimilating sulfate reducing genes | |
| K00394 | adenylylsulfate reductase, subunit A | **K00860** | adenylylsulfate kinase |
| K00395 | adenylylsulfate reductase, subunit B | **K00390** | phosphoadenosine phosphosulfate reductase |
| K11180 | sulfite reductase, dissimilatory-type alpha subunit | **K00381** | sulfite reductase (NADPH) hemoprotein beta component |
| K11181 | sulfite reductase, dissimilatory-type beta subunit | **K00957** | sulfate adenylyltransferase subunit 2 |
| K00958 | sulfate adenylyltransferase subunit 3 | **K00392** | sulfite reductase (ferredoxin) |
|  |  | **K00380** | sulfite reductase (NADPH) flavoprotein alpha-component |
|  |  | **K00956** | sulfate adenylyltransferase subunit 1 |
